# Correspondence

**Published:** 1895-09

**Authors:** 


### CORRESPONDENCE.

### WHY NOT MAKE THE VETERINARY DEGREES UNIFORM WITH THE ACADEMIC DEGREES? A SUGGESTION FROM A NON-VETERINARIAN.

EDITOR OF JOURNAL OF COMPARATIVE MEDICINE, ETC.

DEAR DOCTOR: Although not a veterinarian myself, the discussion which is being carried on in the veterinary journals upon the subject of veterinary degrees has interested me considerably, and, even at the risk of laying myself open to the charge of meddling with affairs which do not concern me, I venture to express my views upon this subject in order to show how the matter strikes one who, although outside the strict confines of the veterinary fraternity, is brought into constant contact with its members, both professionally and personally, and who is as anxious as any veterinarian to see the fraternity united and raised to the highest standard.

As a fundamental principle I think we can all unite upon the grounds that, first of all, the veterinary degrees should, so far as possible, correspond with the degrees in other lines of work; also that the degree should be a Latin degree, both as an expression of its scholastic value and in order to make it uniform with other degrees.

Let us look for a moment at the degrees in other lines of study. When a student completes a prescribed course of study in an academic college he receives the degree, *bachelor* of arts, or science, or philosophy, etc., as the case may be. If he continues his work as a post-graduate for one year, or, as is the case unfortunately in many colleges, even if he does not continue his studies, he receives the degree of *master* a year after the degree of bachelor. If he continues post-graduate work for two or three years and then submits a thesis covering original work and passes a rigid examination, he receives the degree of *doctor*.

What do we find now among the veterinarians? We not only find veterinary colleges conferring the degree of *doctor* upon men who have taken a prescribed course of study for only two years, without ever having made a single original observation in their work, or without ever having added an iota to the advancement of their profession, but still worse, we find men who have received the degree of *veterinary surgeon*, etc., but have never received the degree of *doctor*, assuming the title of *doctor* and acting the lie.

First and foremost, I wish to enter a protest against this assumption of the title *doctor* by men who have not received that degree. A *V.S.* has no more right to use the title *doctor* than he has to use the title *senator* or the *A.B.* or *B.S.* has to use the title *doctor of philosophy* or *doctor of science*, and these title-kleptomaniacs are simply lowering themselves in the estimation of the educated classes by their presumptuousness. Suppose every graduate of Yale, Harvard, Princeton, Wesleyan, Amherst, Georgetown, and other colleges who received an *A.B.* should dub himself "*Doctor!*"

How would I obviate this difficulty? By simple principles of honesty. Let every man pose for exactly what he is. If he is a *Mr.* let him use that title, and in reality it is as good a title as any other. If he is a *Dr.* let him use that title with dignity and modesty, and not consent to be "Doc" (ked).

To unify the veterinary degrees of this country is no simple matter, for we find degrees conferred for different amounts of work Looking over the general field, however, the following occurs to me as being a just arrangement, and I do not see that it would be very difficult to carry it out:

To the student who takes a two years' course at an *agricultural college* and brings in veterinary medicine as a secondary branch, no degree in veterinary medicine should be given, for his veterinary studies are incidental. If, on the other, hand he takes veterinary medicine as *major work*, he is entitled to a degree in that subject, and as such degree I would suggest simply "Veterinarian," the Latin form being *Veterinarius* (cf. Columella, a writer on husbandry, A.D. 50).

For the student who is graduated from a veterinary college after a prescribed course of study I would suggest the degree of "Bachelor of Veterinary Arts" (V.A.B.), the Latin form of which would be *Veteriuariarum Artium Baccalaureus.* This degree would thus correspond to the degrees A.B., Ph.B., S.B. (or B.A., etc.), conferred by academic institutions.

For the V.A.B. who takes a post-graduate course of one year at a veterinary college and then submits a thesis, or to the V.A.B. who completes two years of practice and then submits a thesis, I would suggest the degree "Master of Veterinary

Arts" (V.A.M.), the Latin form of which would be *Veterinariarum Artium Magister.* This degree would correspond to the Master of Fine Arts (*Bonarum Artium Magister*) of the academic colleges.

For the V.A.M. who completes three years of post-graduate work at a veterinary college or a university (*i. e.*, two years more than is required for the V.A.M.), and who then submits a thesis covering original investigation and passes a rigid examination, or who has spent six or eight years in practice after graduating with his V.A.B., and then submits a thesis covering original work and passes a rigid examination, I would suggest the degree of "Doctor of Veterinary Arts" (V.A.D.) *Veterinariarum Artium Doctor*, which would thus correspond to the degrees "Doctor of Philosophy" and "Doctor of Science" conferred by universities.

The expression "*Veterinariarum Artium*," which I have used above in the plural, is open to some objection on account of its length, and it would perhaps be better to use the singular *Veterinariæ Artis*, which would be admissible, since the singular is sort of a collective noun and would include all the branches of science requisite for the veterinarian. The three degrees would then read:

*Veterinariæ Artis Baccalaureus.*

*Veterinariæ Artis Magister.*

*Veterinariæ Artis Doctor.*

In the foregoing scheme I have followed the plan of the academic rather than the medical degrees, as the title Doctor of Medicine in this country represents so many different degrees that it is practically simply means that the man who has it has been graduated at a one, two, three, or four-year medical school. The academic degrees of the country are, on the other hand, much more uniform. Furthermore, the Doctor's degree conferred by American medical colleges is not equivalent to a *Doctor's* degree at all, but is in reality a *Bachelor's* degree, and I believe the degrees of medical colleges need a revision almost as badly as the degrees of veterinary colleges. As I am here discussing veterinary degrees, however, I will not enter further upon the subject of the medical degrees.

It may be a matter of surprise that I suggest the term Veterinary Arts, instead of Veterinary Medicine or Veterinary Science. The suggestion, however, meets several points which have given rise to discussion and disagreement heretofore; some persons have claimed that surgery is not medicine, and on that account objected to the title "Veterinary Surgeon," while others submit that medicine, strictly speaking, does not include surgery, and hence object to the combination "Doctor of Veterinary Medicine." While I consider both of these views extreme, the combination "Veterinary Arts" would offer a middle ground upon which all could probably unite. Furthermore, the practice of surgery and medicine is in reality an art, a practical application of various sciences to a given object, and is not of itself, strictly speaking, a science, on which account I object to the degree "Doctor of Veterinary Science." There are indeed veterinary sciences (which are the same as the medical sciences), *i. e.*, chemistry, physics, zoölogy, botany, etc., but, strictly speaking, there is no "Veterinary Science" *per se*.

Precedent exists in this country for the use of the word art in medical degrees, for upon a diploma which I have recently seen, awarded by the Medical School of the University of Pennsylvania, I noticed the expression *Doctorem in Arte Medica*.

Some of the gentlemen who have been discussing the subject of veterinary degrees forgot to consult their lexicons, and their memories have led them into slight errors regarding the Latin forms of the word Veterinarian, etc. In order to clear up this point I take the liberty of giving the various Latin words for which authority exists. *Veho, vehere, vexi, vectum*, a verb of the third conjugation, means "*to bear, carry, convey*, on the shoulders, by wagon, by horse, by ship, etc." From this verb we have the adjective *veterinus, a, um* (cf. Sext. Pompeius Festus-a grammarian about (?) A.D. 150), which means "of or belong, ing to carrying or drawing burdens." With *bestia* (Latin for beast, as a being without reason; opposed to man; while animal—a living being, includes man) it is translated "a beast of burden or draught." The feminine substantive *veterinæ, arum*, and the neuter substantive *veterina, orum*, refer directly to "draught-cattle," "beasts of burden" (cf. Varro, a writer on husbandry, B.C. 27, and Plinius, a naturalist, A.D. 79).

The adjective *veterinarius, a, um*, means "of or belonging to beasts of burden and draught." Combined with the feminine noun *medicina*, it means "farriery" (cf. Columella, A.D. 50).

In combination with the Latin *ars, artis*, feminine ["skill in producing any material form, handicraft, trade, occupation, employment," and, "with the idea extended, any physical or mental activity, so far as it is practically exhibited, a profession, art, (music, poetry, medicine, etc.")], it means "the art of healing domestic animals;" cf. P. Vegetius (A.D. 420), *De Arte Veterinaria sive de Mulomedicina.*

As substantive *veterinarius* (*ii*, masculine) means "a cattle doctor, farrier, veterinarian" (cf. Columella). *Veterinarium* (*ii*, neuter noun), however, means "a place for taking care of diseased animals;" cf. Hyginus (Gromat.), writer on surveying, A.D. 100.

From this explanation of the Latin terms, which anyone can verify if he will look them up in a Latin dictionary (Harper's, for instance), it will be seen that the combination, "Veterinary Art," can be traced back to A.D. 420, when Vegetius used it in the sense I propose it in the veterinary degree. "Veterinary medicine" is a much older combination, but, according to Harper's *Dictionary*, Columella (A.D. 50) used it referring to "farriery."

The plan I propose above is a radical change from the present veterinary degree, I admit, but some radical change is evidently called for.

The question naturally arises, What are all of the men to do who already have degrees? A most simple solution and one to which I cannot see the slightest objection would be for the colleges which conferred these degrees to give a new diploma with the new degree upon surrender of the old diploma, a method of procedure for which precedent exists.

The foregoing suggestions are made with all due modesty, but, as I do not possess a veterinary degree of any kind, I think I can claim they are not based upon partiality, but simply upon a friendly desire to make veterinary degrees correspond with other degrees and to have the veterinary degree carry some definite meaning with it.

Very respectfully yours,

CH. WARDELL STILES, A.M., PH.D.

WASHINGTON, D. C., July 23, 1895.

---

The greatest dog-owner in the world is Gustav Javanovitch, the cattle-king of the Russian steppes, who employs thirty-five thousand shepherd dogs of various breeds for his one million five thousand sheep.

Preparations for the first examination in New York State are nearly completed.

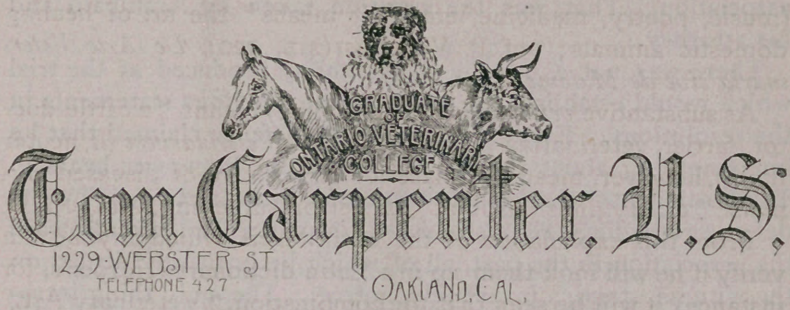

EDITOR JOURNAL OF COMPARATIVE MEDICINE, ETC.

DEAR SIR: I find in your June number of the JOURNAL an anonymous communication concerning myself? I might be justified in treating that article with the contempt that anonymous articles deserve and usually receive. If the writer believes that he is telling the truth, why should he withhold his name? But an anonymous communication when given to the entire profession by a publication so well known as THE JOURNAL OF COMPARATIVE MEDICINE AND VETERNARY ARCHIVES, may do considerable injustice. I therefore make a refutation of the false statements and innuendos contained in that article and desire that it be given as wide a publicity as the article itself has had.

The article contains one or two statements that are founded in fact, but the tenor of the whole is misleading. I have already answered the article contained in the April number, and have shown the falsity of its statements and the meanness of the spirit which actuated the making of them.

From reading the anonymous communication it would appear that Dr. Carpenter was being tried instead of Dr. Archibald. How could the trial "be deemed an auspicious occasion to expose Dr. Carpenter" when Archibald's entire defense was based upon the plea that he was not the author of the statements contained in the resolutions which libelled Dr. Carpenter? He claimed that he was only acting as the agent of the association in writing and sending out the resolutions; the whole matter was hearsay with him; that he knew nothing personally as to the truth of the statements contained in the resolutions. The jury acquitted him because they thought that an officer of an association who should write and send out resolutions passed by the association should not be punished for the acts of the association. That was the position taken by Archibald and his attorney.

There was not a particle of testimony produced at the trial which would establish the truth of the libellous statements in the resolutions. Every witness for the defense claimed that he knew nothing about the matter, that all he knew was hearsay. The statement "that much testimony was introduced by the defense regarding the position Dr. Carpenter had taken toward the association in the past, all of which he positively denied on the witness stand," is entirely untrue. The only thing introduced in evidence regarding my position in the past toward the association was the article which was published in the January number, 1889, of the *American Veterinary Review* over my signature. The further statement in the June article, that "he even denied having anything to do with a protest against a certain bill in the Legislature," is correct. That protest was not scandalous, as he says, but foolish. My name was appended to it in print without my knowledge or consent, and I never signed the protest. There would be nothing in the act of signing it except that the protest was foolish and useless. There was no attempt made at the trial to show that the protest was written over my signature. The protest was not introduced at the trial, because it was immaterial and irrelevant, and was not offered in evidence. I was merely shown a printed copy of the protest and asked if I had signed it. This anonymous writer must have known that the statement inferentially made in his communication, "that it was seldom that a man had his character exposed as was the character of the prosecuting witness," was absolutely untrue. There was no attempt to attack my character; the defendant had all he could do to protect his own. This writer closes up his article by making the following statement: "It might also be interesting to state that Dr. Carpenter did not gain the position of meat and milk inspector of the city of Oakland." That is true. But why make the statement? I never was an applicant for the position. He might with as much propriety say that I was not appointed inspector for the city of New York. The spirit of the anonymous article is unfair and unjust, and could only be written by one who dared not attach his name to the article.

T. CARPENTER, V.S.

JULY 22, 1895.

